# Stepping outside the clinic walls: health professionals’ encounters with place during the COVID-19 pandemic

**DOI:** 10.1186/s12889-026-27092-y

**Published:** 2026-03-26

**Authors:** Ayşe Polat, Zübeyde Demircioğlu, Hüseyin Küçükali

**Affiliations:** 1 Department of Sociology, Boğaziçi University, İstanbul, Türkiye; 2 Department of Sociology, İstanbul Medeniyet University, İstanbul, Türkiye; 3 Queen’s University Belfast, Centre for Public Health, Belfast, BT12 6BA UK; 4 Research Center for Healthcare Systems and Policies, İstanbul Medipol University, İstanbul, Türkiye

**Keywords:** Health professionals, COVID-19, Contact tracing, Türkiye, Social determinants of health, Health inequalities, Place

## Abstract

**Background and aim:**

While existing literature has largely examined healthcare delivery within hospitals during the COVID-19 pandemic, this study extends the inquiry to the spatial dimensions of public health work in urban settings. It explores the experiences of health professionals engaged in home-based contact tracing in Türkiye, focusing on their perceptions of social determinants of health through place.

**Methods:**

Semi-structured interviews and focus group discussions were conducted with 45 health professionals from socioeconomically diverse districts in Istanbul, between July 2021 and September 2022. Data were analyzed using thematic analysis, with particular attention to the role of place.

**Results:**

Health professionals observed a range of administrative, social, economic, cultural, and environmental challenges across households and neighborhoods they visited. Three key themes were identified: challenges in navigating the city (urban barriers and contrasting levels of urban development), perceived administrative neglect of place (time-pressured, place-ignorant planning and unregistered places), and places unsettling medical practice (socioeconomically deprived households and perceived limits of biomedicine in sociocultural contexts). These findings illustrate how place influenced health professionals’ experiences and highlight the underappreciation of place as a crucial social determinant of health.

**Conclusions:**

This study underscores the role of place in shaping health professionals’ perceptions of social determinants of health during COVID-19 contact tracing in Türkiye. As their awareness of health inequalities increased, health professionals experienced moral distress arising from tensions between biomedical training and complex, non-medical needs. While socioeconomic constraints were often interpreted sympathetically, sociocultural factors were more frequently moralized, pointing to gaps in cultural competence. Taken together, these findings demonstrate how place-based public health work reveals the limits of a purely biomedical approach and underscore the importance of systematically integrating health professionals’ experiential knowledge into place-sensitive, culturally competent, biosocial approaches to health policy and practice during emergencies and beyond.

**Supplementary Information:**

The online version contains supplementary material available at 10.1186/s12889-026-27092-y.

## Introduction

COVID-19 was a major global crisis, causing millions of deaths and widespread disruption to socioeconomic structures and everyday life, prompting countries to respond through a range of public health strategies. During the early phases of the COVID-19 pandemic, before vaccines became widely available, contact tracing emerged as a key non-pharmaceutical intervention aimed at interrupting transmission. Although contact tracing had been used in previous outbreaks like smallpox, Ebola, and SARS, its scale and intensity during the COVID-19 pandemic were unprecedented [1]. Countries implemented contact tracing strategies in diverse ways, ranging from digital tracing technologies to targeting specific vulnerable groups [2, 3].

Türkiye’s pandemic response was distinctive in its reliance on home-based contact tracing carried out by health professionals. This strategy shifted health professionals from their accustomed roles of providing biomedical care within hospitals to visiting patients’ homes in the urban environments where they lived. Organized by provincial and district health directorates, contract tracing teams included doctors, nurses, allied health professionals, and drivers. They visited individuals who tested positive for COVID-19, collected information about recent social interactions, and communicated isolation and prevention measures. Implemented at a large scale, this home-based model positioned contact tracing as an out-of-hospital practice that routinely brought health professionals into direct contact with the social, economic, and environmental conditions shaping patients’ lives, creating opportunities to observe health disparities.

To examine these encounters, the study draws upon a biosocial approach that emphasizes the mutual constitution of biological and social factors and the interplay between human and socioenvironmental elements in shaping health and illness [4]. Within this framework, the concept of social determinants of health highlights how non-medical factors– such as economic conditions, education, housing, and neighborhood environments– influence health outcomes [5] and can contribute to health disparities and health inequities [6, 7]. Although these inequalities predated COVID-19, the pandemic intensified existing vulnerabilities [8].

Place plays a significant role as a social determinant of health. Encompassing both natural and built environments, the place directly and indirectly influences health outcomes [9]. It is not merely space or location, but a complex concept [10] embedded in material forms and shaped by cultural and social interpretations [11]. Place has been defined as “space imbued with meaning, shaped by social and political-economic forces, and the site for human and non-human interactions” ([11], p. 55).

Since the early 1990s, research across various disciplines has increasingly explored the relationship between health and place. Early studies focused on compositional approaches that examined individual characteristics, such as income and education, while later contextual approaches investigated the broader geographical and socioeconomic characteristics of neighborhoods or regions [12]. More recent relational approaches emphasize the dynamic relationship between personal traits and the broader contextual factors that shape health outcomes [13].

Building on this, public health studies have examined how place-based factors such as access to green spaces, food sites, transportation infrastructure, and exposure to pollution contribute to urban health disparities [14]. Neighborhood deprivation and social vulnerability indices demonstrate how place intersects with socioeconomic status, further influencing health outcomes [15].

However, during COVID-19, the place has received limited scholarly attention beyond epidemiological concerns and has rarely been examined as a socially and materially embedded context through the perspectives of health professionals. While place-based inequalities in exposure and outcomes have been addressed to some extent, far less is known about how frontline health professionals themselves encountered, interpreted, and navigated these environments.

Despite the importance of contact tracing in pandemic response, existing research has largely focused on quantitative assessments of effectiveness, digital strategies, or cross-national comparisons of contact tracing policies [16–18]. The experiences of health professionals involved in contact tracing, particularly in non-hospital and home-based settings, have received less scholarly attention. While hospitals have been the focal point in much of the pandemic research [19, 20] and numerous studies have examined the challenges health professionals face in hospital settings [21, 22], their encounters with patients’ households and neighborhoods through contact training remain underexplored.

This study foregrounds place as the primary analytic lens through which health professionals’ accounts are examined. Focusing on home-based COVID-19 contact training in İstanbul, Türkiye, it explores how visits to patients’ homes and neighborhoods enabled health professionals to observe the living conditions of households firsthand, leading them to perceive place not merely as a physical location but as a socially structured environment that can be health-promoting or health-limiting. While the pandemic constituted a major moment of crisis that revealed existing vulnerabilities, it also created conditions under which health professionals could recognize non-medical barriers to healthcare, reflect on structural inequalities, and articulate strategies of resilience and response.

Accordingly, this study addresses the following research questions:

1. How did health professionals experience patients’ households and neighborhoods while conducting home-based COVID-19 contact tracing at different districts of Istanbul, Türkiye?
2. How did the place-based encounters shape their perceptions of social determinants of health and health disparities?
3. How did these experiences prompt reflection on professional practice, including perceived roles, capacities, and the limits of biomedical intervention?

## Methods

### Study design

This study was designed as qualitative research to explore health professionals’ experiences during COVID-19 contact tracing in Istanbul, Türkiye. Qualitative methodology is particularly suitable for examining individuals’ subjective interpretations, including their thoughts, feelings, and perspectives regarding a lived phenomenon [23]. The focus of this study is on health professionals’ perceptions of place-based, social, environmental, economic, and cultural determinants of health encountered during home-based contact tracing.

### Data collection

Contact tracing teams in Türkiye were organized by the provincial and district health directorates. To recruit voluntary participants, district health directorates in Istanbul were contacted. The intense workload of contact tracing limited responses; nevertheless, a sufficient purposive sample was achieved, representing diversity in professional roles, institutional positions, and district context among health professionals deployed in contact tracing. Data collection proceeded until thematic saturation was reached, defined as the point at which additional interviews or focus group discussions no longer generate substantively new themes relevant to the research questions.

Data were collected through semi-structured interviews and focus group interviews. Semi-structured interviews were conducted with health professionals between July and October 2021, and focus group interviews were held in September 2022. In total, 18 participants from various age groups, genders, and health professions, as well as 3 drivers who transported teams across the city, participated in the semi-structured interviews. An interview form was developed by the research team to ensure consistency across interviews while allowing flexibility for participants to elaborate on their experiences. The interview guide is provided in the appendix (Supplementary Material).

For the focus group interviews, every district health directorate in Istanbul was invited via both phone calls and emails to increase participation. Two focus group sessions were conducted, consisting of 13 and 14 participants. District-level diversity was a key consideration in the sampling strategy, as socioeconomic conditions vary substantially across Istanbul. While semi-structured interviews were conducted with participants from mainly two districts, focus group interviews included participants from 14 districts out of a total of 39 districts in İstanbul. Districts represented in the study spanned the full range of socioeconomic status (SES) categories, from the most affluent to the most socioeconomically deprived. It is important to note that socioeconomic conditions also vary within districts at the neighborhood level; therefore, district-level classification is used here as a contextual indicator rather than a precise measure of participants’ lived environments.

Details about interview participants are presented in Table 1. Individual-level demographic characteristics such as age were not systematically collected for focus group participants, as focus group interviews were designed to foreground collective experiences and feedback on initial findings presented to participants. Corresponding information for focus group participants is presented in Table 2.

**Table 1:** Characteristics of semi-structured interview participants

Participant	Gender	Age	Profession	Contact tracing	District
P1	Female	20–29	Doctor	> 12 months	Üsküdar
P2	Female	30–39	Nurse	6–12 months	Üsküdar
P3	Male	20–29	Doctor	6–12 months	Eyüpsultan
P4	Female	30–39	Midwife	> 12 months	Eyüpsultan
P5	Male	30–39	Doctor	> 12 months	Eyüpsultan
P6	Female	20–29	Doctor	6–12 months	Eyüpsultan
P7	Male	NA	Social worker	< 6 months	Eyüpsultan
P8	Male	20–29	Doctor	6–12 months	Eyüpsultan
P9	Male	30–39	Dentist	> 12 months	Eyüpsultan
P10	Female	20–29	Doctor	6–12 months	Üsküdar
P11	Male	40–49	Driver	> 12 months	Üsküdar
P12	Male	20–29	Dental technician	6–12 months	Üsküdar
P13	Female	30–39	Dentist	6–12 months	Üsküdar
P14	Male	30–39	Driver	< 6 months	Üsküdar
P15	Female	20–29	Technician	> 12 months	Üsküdar
P16	Male	30–39	Driver	> 12 months	Eyüpsultan
P17	Male	30–39	Health director	> 12 months	Eyüpsultan
P18	Male	30–39	Health director	> 12 months	Üsküdar
P19	Female	40–49	Nurse	6–12 months	Kadıköy
P20	Male	20–29	Doctor	> 12 months	Kadıköy
P21	Female	30–39	Health director	> 12 months	Fatih

**Table 2:** Characteristics of focus group interview participants

Participant	Gender	Profession	District
P1	Female	Doctor	Başakşehir
P2	Female	Psychologist	Başakşehir
P3	Female	Doctor	Beşiktaş
P4	Female	Health director	Beşiktaş
P5	Male	Doctor	Beşiktaş
P6	Female	Doctor	Beyoğlu
P7	Male	Doctor	Beyoğlu
P8	Male	Social worker	Esenyurt
P9	Male	Radiology technician	Esenyurt
P10	Male	Doctor	Esenyurt
P11	Female	Doctor	Esenyurt
P12	Female	Doctor	Fatih
P13	Female	Doctor	Kağıthane
P14	Male	Doctor	Kağıthane
P15	Female	Doctor	Sancaktepe
P16	Female	Doctor	Sancaktepe
P17	Male	Doctor	Sarıyer
P18	Male	Doctor	Sarıyer
P19	Female	Doctor	Sultanbeyli
P20	Female	Doctor	Sultanbeyli
P21	Male	Health director	Sultanbeyli
P22	Male	Doctor	Sultangazi
P23	Male	Health technician	Sultangazi
P24	Female	Doctor	Sultangazi
P25	Female	Doctor	Tuzla
P26	Female	Doctor	Ümraniye
P27	Male	Health director	Üsküdar

In addition to generating new data, the focus group sessions functioned as a form of member checking aimed at enhancing the credibility of the findings ([24], pp. 315–316). Participants were presented with preliminary themes derived from the interviews and invited to reflect on their accuracy, completeness, and resonance with their own experiences ([23], p. 209). In line with Morgan’s argument that the level of formal structure in focus groups should be determined by the goals of the research rather than a rigid protocol, the discussions prioritized collective reflection on emerging findings rather than strict adherence to a standardized question sequence [25].

Şeker et al. [26] examined the socioeconomic status (SES) of neighborhoods within Istanbul’s districts, ranking them from A to E (highest to lowest). Based on their data, the mean SES category for each district was calculated and is shown in Table 3. The C-level SES, which indicates the mean, was the most common category in this research’s sample. The sample included participants from districts across all SES levels, A (the most affluent) through E (the most deprived). Table 3 shows the district information pertaining to all participants, both interview and focus groups.

**Table 3:** Characteristics of participants and districts

District	Mean SES (2016)	Population (TURKSTAT, 2021)	Area (km²) (DGM, 2014)
Başakşehir	C	503,243	107
Beşiktaş	A	178,938	18
Beyoğlu	C	233,322	9
Esenyurt	D	977,489	43
Eyüpsultan	C	417,360	228
Fatih	C	382,990	15
Kadıköy	A	485,233	25
Kağıthane	C	454,550	15
Sancaktepe	D	474,668	63
Sarıyer	B	349,968	177
Sultanbeyli	E	349,485	29
Sultangazi	E	543,380	37
Tuzla	C	284,443	138
Üsküdar	B	525,395	35

### Data analysis

Data analysis was conducted collaboratively by the research team using ATLAS.ti software. Each researcher coded the first five interviews independently, after which the first author developed a preliminary codebook incorporating codes generated by each researcher. The research team revised the codebook multiple times to enhance reliability and agreement [27].

Thematic analysis was carried out following the principles outlined by Braun and Clarke [28]. This process involved data familiarization, assignment of codes, identification of patterns, review of themes, and finalization of the report. Through inferential logic, data were classified by similarity [29], and through inductive and comparative reasoning, differences were identified, leading to the development of themes and subthemes [30].

While the analysis process involved categorization, close attention was paid to maintaining narrative coherence and analytical proximity to the main research question [31]. Alternative explanations and different conceptualizations were continuously assessed to enhance the integrity, nuance, and depth of the qualitative analysis [32].

Themes were identified to “bring meaning and identity to a recurrent experience and its variant manifestations” ([33], p. 362). They were developed to capture recurrent patterns of meaning in health professionals’ accounts of place and social determinants of health, while also attending to variation and complexity in experiences across interviews and focus group discussions. With regard to the themes reported in this article, participants largely converged in recognizing that place-based conditions shaped both pandemic transmission and the practice of contact tracing. While contrasting perspectives emerged in other parts of the broader project [34], the data analyzed here primarily reflected shared professional observations about place-based inequalities rather than substantial disagreement.

To protect participants’ anonymity, only profession, age, and district information are provided in the results sections. For health directors, however, district information is omitted to further safeguard their identity and prevent potential disclosure.

**Fig. 1:**
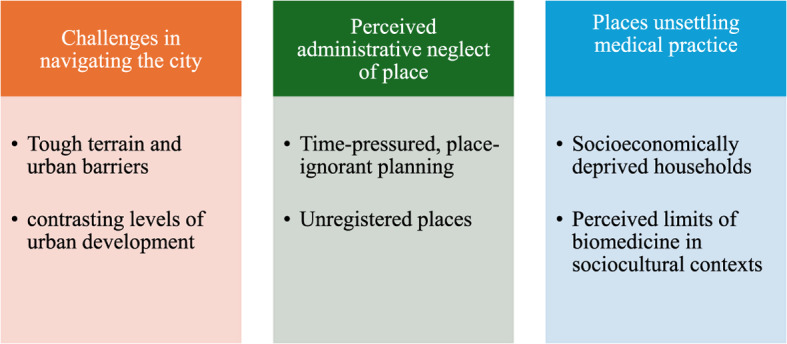
Themes and subthemes

The themes and subthemes generated through this analytical process are summarized in Fig. 1.

## Results

In this study, three main themes, each with two subthemes, were identified in health professionals’ observations of social determinants of health during contact tracing (Fig. 1). These themes capture participants’ accounts of how place shaped both their working conditions and their perceptions of health inequalities.

### Challenges in navigating the city

While navigating the city, health professionals occasionally encountered “very beautiful landscapes” that prompted them to pause and take photographs (dental technician, Üsküdar). However, amid the intense workload of the pandemic, their experience of urban space was shaped primarily by practical challenges related to İstanbul’s topography, infrastructure, urban development, and administrative organization.

#### Tough terrain and urban barriers

Health professionals described certain locations as inaccessible or difficult to access due to challenging topography and inadequate urban planning. The rough terrain of Istanbul–with its hills, steep slopes, winding roads, and curved ramps–was frequently mentioned as a major obstacle and vividly recounted as an experience likely to remain imprinted in memory:

“I do not think I will ever forget the roads of Eyüp. Go up and down the hill, I cannot forget those roads. We were continuously in the car.” (doctor, female, 26, Eyüpsultan)

Participants reported that squatter settlements, narrow and car-inaccessible streets, and missing house and street numbering further complicated efforts to locate and reach designated contact tracing sites. These conditions often required health professionals to cover distances on foot, contributing to physical exhaustion:

“In Rami [Eyüp], the streets are very narrow. Traps exist on the road. Cars cannot drive through some streets, so you just have to walk.” (doctor, female, 26, Eyüpsultan).

Such conditions added to the physical strain of an already demanding job, slowing the efficient completion of contact tracing tasks.

Dense traffic further exacerbated these difficulties. Participants associated long car journeys with back pain, headaches, and nausea (dentist, female, 30, Üsküdar; radiologist, female, 25, Üsküdar), and emphasized that traffic congestion delayed timely visits to households despite prompt test results:

“Even though the test results were available by noon, our teams sometimes could not reach those houses until 5 pm due to heavy traffic in Kadıköy.” (nurse, female, 43, Kadıköy).

#### Contrasting levels of urban development

Health professionals described encountering contrasting levels of urban development in housing and neighborhood environments. Under-resourced housing was often physically difficult to access, while affluent areas were characterized by gated or enclaved residences that restricted entry. Participants emphasized that districts frequently contained a mix of both forms of housing. Very poor housing was found alongside wealthy “luxurious houses” (doctor, Eyüpsultan), and “houses in very bad conditions” were located right behind “very beautiful places” (doctor, male, Sarıyer). Each type of housing presented distinct challenges for contact tracing.

Underdeveloped, improvised, or partially constructed buildings lacking basic amenities such as electricity (social worker, Eyüpsultan), as well as older multi-story houses without elevators, were described as physically demanding:

“There were sometimes no elevators, so we had to climb five or six flights of stairs, wearing face masks, and then inform patients about [the COVID-19] again and again while gasping for our breath.” (doctor, male, 24, Eyüpsultan).

Participants also described difficulties navigating gated or enclaved residential buildings, reporting being denied entry by security personnel (nurse, female, 43, Kadıköy) or losing their way out in these exclusive residential buildings:

“We got into enclaved buildings but then could not find the way out; we sometimes got lost in them, really [laughs]. We had to ask security [for directions]. Also, sometimes the security did not let us in.” (midwife, female, 33, Eyüpsultan)

In addition to physical barriers, participants highlighted safety concerns, particularly in poorly lit or deserted areas during late-night shifts, describing unpredictable risks that often left little time to contact the police:

“We used to work until 11:30 pm. In winter, the streets are dark by 6 pm. We went to various places, some without lights and some deserted. Sometimes a dog or a person with a substance addiction would suddenly appear in front of us. A friend of mine fell down the stairs and broke her leg because it was so dark. In some places, there were only streetlights; in others, there were not any at all.” (dentist, female, 30, Üsküdar)

The need for contact tracing teams to enter apartment buildings further heightened safety concerns. One participant recounted an incident where a building door was locked behind them. Although she was unharmed, this experience led the health directorate and their team members to decide they would no longer enter buildings alone (nurse, female, 32, Üsküdar). Participants also reported verbal and physical hostility from residents who rejected the COVID-19 and isolation measures, adding another layer of insecurity to their work environment.

### Perceived administrative neglect of place

Health professionals described administrative arrangements that, in their view, failed to adequately consider the socioenvironmental features of place in the management of contact tracing. They underlined that this oversight complicated the contact tracing process.

#### Time-sensitive, place-ignorant planning

Health professionals described a conflict between bureaucratic and epidemic spatialities, pointing to a mismatch between administrative arrangements and the dynamics of epidemic spread. They noted that many districts in Istanbul covered extensive geographic areas, and that the bureaucratic assignment of cases to contact tracing teams strictly on administrative boundaries was often inefficient.

They emphasized that neighborhoods located at the edges of a district were sometimes geographically closer to neighborhoods in adjacent districts than to other areas within their own district. From their perspective, this misalignment rendered the division of contact tracing work based solely on administrative boundaries and timeliness criteria ineffective.

“Eyüp covers a vast area, and the distances between cases are substantial. For example, Akpınar is in Eyüp, but it takes two and a half hours to get there. For a team to arrive and see one case, it takes about 3 hours. Yet, in another district, in 3 hours, you can see maybe up to 50–60 cases.” (doctor, male, 25, Eyüpsultan)

Health professionals proposed that it would have been much more effective to visit cases located close to one another within the same neighborhood, rather than traveling between distant neighborhoods (dentist, female, 30, Üsküdar; doctor, female, 26, Eyüpsultan).

Participants further noted that the health bureaucracy’s emphasis on timeliness–requiring contact tracers to reach cases as quickly as possible regardless of location– ignored the spatial organization of urban life, suggesting that a spatial optimization algorithm, prioritizing proximity and clustering of cases rather than solely elapsed time since diagnosis would have reduced time spent in traffic, minimized long-distance travel, and enabled teams to visit more households per day.

#### Unregistered places

Health professionals described difficulties in locating and tracking residents in unregistered places, pointing to high population mobility, delays in updating citizens’ contact information, and technological limitations as key challenges. Participants described difficulties locating both immigrants (midwife, Eyüpsultan) and Turkish citizens at their registered addresses. They emphasized that frequent movement across neighborhoods and districts, combined with outdated population address records, created persistent challenges:

“As a society, we are constantly on the move. People rarely update their addresses when they move to new locations. Population records are outdated.” (doctor, male, 38, Eyüpsultan)

To locate correct addresses, contact tracing teams sought assistance from the Ministry of Interior and other departments, at the cost of additional time and administrative effort.

Technological limitations compounded the problem. Health professionals emphasized that poor internet and phone signal coverage posed significant challenges. Health directorates required contact tracers to enter a code to verify their presence at specific addresses. However, weak phone signals and inadequate digital infrastructure often prevented accurate location verification (doctor, male, Beyoğlu).

### Places unsettling medical practice

Health professionals described being both professionally and personally affected by the socioeconomic, demographic, and cultural characteristics of the households they visited during contact tracing. They emphasized that these encounters with non-medical factors heightened their awareness of the limits of biomedical approaches.

#### Socioeconomically deprived households

Health professionals described witnessing patients’ living conditions firsthand as one of the most challenging aspects of home-based contact tracing. Some participants reported being so affected by the desperate conditions they encountered that they experienced emotional distress, including tears and nervous breakdowns (nurse, Üsküdar). To cope, many turned to social services for support (nurse, female, 43, Kadıköy; health director).

Many health professionals reported encountering extreme poverty firsthand for the first time in their personal and professional lives, describing the experience as deeply shocking (dentist, female, 30, Üsküdar):

“We entered a house where the auntie, unable to walk, was alone and couldn’t clean or cook. The strong odor was noticeable even through two masks. The walls were crumbling, the furniture was ruined, and the kitchen was a total mess. I have never seen places like this before.” (doctor, female, 28, Üsküdar)

While these living conditions were described as shocking, participants also raised ethical dilemmas related to their professional roles. They described struggling to reconcile their biomedical training with the realities of patients’ circumstances, noting that their guidance on room separation and self-isolation was often impractical:

“We tell people, ‘Separate your room, and if possible, your bathroom, too.’ But I felt ashamed to say this. Only newer houses have a separate bathroom in the master bedroom. Yet, in many of the places I visit, people are living in just one room. When the door opens, I see five people sleeping in the same bed. What can I say to them?” (dentist, female, 30, Üsküdar)

Health professionals also described how recommendations regarding healthy meals and vitamin intake were often unrealistic for low-income households:

“Healthy meals and vitamins are important, but can people afford them? As contact tracers, we leave medicine at the front door. When the door opens, you can immediately sense the household’s conditions. Can they afford vitamins? Can they buy fruits and vegetables? Some families need financial support to afford these.” (midwife, female, 33, Eyüpsultan)

Participants further highlighted how economic insecurity, particularly among daily wage earners, complicated adherence to public health guidelines:

“One of the biggest problems was people who earned a daily income. They would tell us not to register them as contacts, asking, ‘How will I put food on the table if you list me as a contact?’” (nurse, female, 32, Üsküdar)

Such cases also generated feelings of uncertainty and distress:

“When we told patients that they needed to isolate at home for 14 days, they would ask, ‘If I do not work for 14 days, how will I make a living this month?’ I did not know what to say to that person.” (doctor, female, 28, Üsküdar)

While such accounts highlighted the constraints faced by economically disadvantaged households, participants did not attribute pandemic transmission exclusively to poverty. Across interviews and focus group discussions, health professionals widely acknowledged that COVID-19 spread occurred across all socioeconomic contexts, although the mechanisms of transmission were perceived to differ—for example, overcrowded housing and economic precarity in deprived areas, and mobility, travel, or social gatherings in more affluent settings (female, doctor, Başakşehir; male, radiology technician, Esenyurt; female, doctor, Sultanbeyli).

#### Perceived limits of biomedicine in sociocultural contexts

Health professionals emphasized that sociocultural factors – including family structures, everyday sociocultural practices, and health beliefs– played an important role in shaping adherence to pandemic regulations, with certain sociocultural contexts presenting more concentrated challenges for implementation.

While underscoring their commitment to protecting public health during the pandemic, health professionals also narrated what they perceived as contradictions in societal behavior. They attributed the persistent rise in cases not only to the nature of COVID-19 transmission but also to sociocultural factors, such as skepticism toward health measures, limited health literacy, and continued attachment to pre-pandemic sociocultural routines, including frequent and crowded family gatherings. While reflecting participants’ genuine frustrations, such accounts tend to frame non-adherence as a matter of cultural disposition rather than structurally conditioned behavior–a pattern explored further in the Discussion.

Health professionals described how certain sociocultural places were experienced as posing increased risks for virus transmission, often due to overcrowded living conditions and everyday social practices–circumstances that prompted repeated contact tracing visits.

“We have come to memorize some street names.” (doctor, male, 38, Eyüpsultan)

Family compounds—where multiple generations or extended family members lived together in close quarters—were frequently characterized by participants as epidemiological risk factors. Participants noted that these living arrangements were more prevalent in certain neighborhoods, leading to repeated contact tracing visits to the same addresses:

“When someone get positive test in a family apartment, then one by one everyone turns positive. We would start contact-tracing with one surname and, within the same week, end up visiting the same address multiple times, seeing a minimum of three or four other individuals from the same family.” (doctor, female, 25, Eyüpsultan)

Health professionals also described difficulties when working with certain ethnic and immigrant populations, often attributing limited compliance with isolation, curfews, and mask-wearing to customs, everyday practices, and lifestyles. Several referred to neighborhoods with larger immigrant populations—such as Syrian, Afghan, or African residents—as contexts where they perceived greater challenges in implementing pandemic-related measures. Such accounts reflect participants’ perceptions of cultural difference as a barrier to compliance, a framing that, as discussed in the Discussion, risks obscuring the structural vulnerabilities shaping these communities’ circumstances.

“Some people, like the Syrians, are very close-knit. They never wear masks at home. Positive-testing cases open the door to us without a mask, and there might be a thousand people inside. I am not sure if they do not know or just do not care?” (midwife, female, 33, Eyüpsultan)

Health professionals also reported encountering skepticism toward COVID-19 in some neighborhoods, which they attributed to cultural beliefs about health and disease prevention:

“Some people just do not believe in COVID-19, or they are opposed to medication and vaccination. It has to do with culture.” (doctor, male, 25, Eyüpsultan)

Health professionals repeatedly emphasized education and health literacy as key factors shaping adherence, pointing to difficulties in understanding medical instructions as major obstacles to compliance. These accounts reflect a tendency to locate non-adherence within individuals’ educational or cultural deficits, rather than considering how structural inequalities — including unequal access to education, information, and resources — shape health literacy and compliance. This framing is examined critically in the Discussion.

“I wish our people were more educated and could adhere to the rules. Our society is very undereducated. We deliver medication to patients, and individuals in their 30s or 40s say, ‘I can’t read.’ Some of these people are also in poor economic conditions, unable to afford even a face mask. And yet, to see such illiteracy… It has been more than a year, and some are still asking, ‘So, what are we supposed to do now?’ Don’t you read or watch the news? Some of them live in very dire conditions, but it all comes down to education.” (dentist, female, 30, Üsküdar)

Several health professionals described encounters with individuals who struggled to follow medical instructions as frustrating and time-consuming. Such accounts again suggest a tendency to attribute difficulties to individual or societal deficits, a framing that is examined further in the Discussion.

“I sometimes had to explain to people how to use the medication ten times. I do not expect everyone to be at the same cultural level, but I do not think using these medications is such a complicated task that I should have to explain it more than ten times to so many people. The education level in society really needs to improve.” (doctor, male, 25, Eyüpsultan)

Health professionals also described encountering forms of social diversity and living arrangements that differed from their expectations. While they frequently associated increased transmission risk with extended family living arrangements, several participants expressed surprise at observing many individuals, particularly older adults, living alone, especially in higher socioeconomic neighborhoods.

“The houses in Bebek, Arnavutköy look very nice from the outside, but inside, many bedridden elderly individuals lived with their [foreign] caregivers. I was stunned to see this. It made me feel sad.” (doctor, male, Beşiktaş)

Health professionals also described challenges related to communication across linguistic and cultural differences. Language barriers were experienced not only in interactions with immigrant populations but also with some local residents who spoke unfamiliar dialects or accents:

“I faced great challenges communicating with people. My father and mother speak different Turkish accents, too. During contact tracing, there were times when I could not understand a single word of what some people were saying.” (doctor, male, 25, Eyüpsultan)

Several participants reported adapting their interaction styles over time, including improving basic communication skills, such as conducting conversations over the phone (doctor, Eyüpsultan; radiology technician, Üsküdar) and adjusting their approach when working with households from diverse socioeconomic and cultural contexts (dental technician, Üsküdar).

## Discussion

Health authorities in Türkiye mobilized diverse health professionals for home-based contact tracing across the country. This provided them with a unique professional experience. During contact tracing, they encountered place as a multidimensional social determinant encompassing topographic, infrastructural, developmental, administrative, organizational, socioeconomic, and cultural dimensions. The place was not simply a background context but actively shaped both the health status of individuals visited and the working conditions and well-being of contact tracing teams. Place, in this sense, was constitutive of their practice, “integral to the very structure and possibility” of their professional experience ([35], p. 31).

Because interpretation is central to the experience of place [35, 36], health professionals ascribed meaning to the environments they encountered through their personal, socioeconomic, cultural, and professional backgrounds. Place is often perceived through dichotomies, such as safe/dangerous, accessible/inaccessible, new/old, familiar/unfamiliar, affluent/deprived [11]. Health professionals constructed similar interpretive categories during contact tracing. They described some places as difficult to navigate due to poor urban planning, inadequate infrastructure, and weak administrative coordination, and expressed astonishment at the sharp contrasts between well-developed and underdeveloped, affluent and deprived neighborhoods.

The findings suggest that the place shaped health professionals’ experience through contrasts along two main axes, followed by cycles of (re)interpretation.

### Transition from clinical to home settings: professional frustration against biosocial realities

*The first axis* was the change of their workplace from clinics, hospitals, and administrative offices to citizens’ homes and neighborhoods. This created a contrast between their centralized, standardized, place-insensitive expectations and the socioeconomic realities of the people. Home visits rendered living conditions directly visible, which were largely absent from routine clinical practice or administrative tasks. It led to a comparison between biomedical and biosocial approaches to health. Despite their disillusionment with the biomedical model and their recognition of biosocial shortcomings in skills, inadequate resources and institutional support limited their ability to respond and created frustration.

Prior research emphasizes the intense pressures faced by health professionals during the COVID-19 pandemic, ranging from heavy workloads and concerns about work-life imbalance to insufficient resources and job insecurity [37–39]. For those engaged in contact tracing, these pressures were compounded by repeated exposure to deprivation and by the mismatch between standardized medical advice and the lived realities of households. Witnessing inequities generated moral distress, a form of psychological discomfort that arises when one’s actions contradict deeply held moral beliefs [40]. Health professionals described tension between their biomedical training and the social realities they encountered, which unsettled their professional self-understandings. Feelings of discomfort, confusion, and even “shame” emerged as professionals became aware of the gap between standardized medical guidance and the socioeconomic and cultural conditions shaping households’ capacities to comply with public health measures.

The urgency of the pandemic intensified this dilemma: health professionals were pressured to act quickly while being unable to address broader structural constraints affecting patients’ well-being. While much of the existing literature on moral distress and ethical challenges faced by healthcare professionals during the COVID-19 pandemic focuses on hospital settings [40–43], this study extends these insights with the experience of health professionals outside clinical environments.

### Transition from one home to another: recognition of determinants of health inequalities

*The second axis* was the change from one patient’s home to another. They encountered the place at multiple levels, including households, buildings, and neighborhoods, as well as the administrative organization of districts. Socioeconomic, ethnic, and cultural factors shaped health professionals’ perceptions of what constituted “healthy” and “unhealthy” places [44]. They observed that individuals living in better-resourced homes were more able to comply with isolation measures and access health-supporting resources, such as nutritious food, vitamins, and adequate living space. In contrast, those residing in smaller, older, or overcrowded apartments, or those working in insecure jobs, were often deprived of these options. Experiencing different places multiple times every day contributed to a deeper recognition of social determinants of health and health inequalities. These observations resonate with Moula et al. [45] and Shostak et al. [46], who show that the COVID-19 pandemic heightened healthcare professionals’ awareness of social determinants, especially during a crisis.

Yet this heightened awareness did not extend evenly across domains: structural constraints were often interpreted sympathetically, whereas sociocultural practices were more readily moralized. Despite recognizing structural influences on health behaviors, many health professionals were more critical when interpreting sociocultural factors. They expressed both explicit and implicit biases [47], particularly toward immigrants and individuals with lower educational attainment, often framing sociocultural practices as matters of personal or communal choice rather than as structurally conditioned behaviors. For example, health professionals suggested that higher education was associated with being more “cultured” or knowledgeable and thus more compliant, while overlooking how educational attainment itself is shaped by unequal access to resources. Non-adherence driven by financial necessity was typically met with more empathy, while resistance linked to cultural attachments was more likely to be interpreted as irresponsible or disruptive to public health efforts.

Similar patterns have been reported in other national contexts. Ahmadi et al. [48] show that in Iran, health professionals identified socioeconomic conditions, housing, and occupation as key factors influencing adherence to preventive guidelines, while cultural values such as fatalism and social customs were seen as barriers. Similarly, Fauk et al. [49] report that in Indonesia, non-adherence was often attributed to limited knowledge, ignorance, or traditional cultural practices. Together, these studies echo the tendency observed in this research to treat sociocultural factors as obstacles to public health measures.

In this study, health professionals did not use overtly discriminatory language, yet their interpretations frequently positioned sociocultural factors as impediments to biomedical implementation. This points to a critical gap in cultural competence, which is the capacity to recognize, respect, and work with patients’ beliefs, lifestyles, and attitudes [50]. As Coombs et al. [51] argue in their study of rural communities in Montana, US, cultural humility–the ability to refrain from imposing one’s own cultural worldview–can be crucial to effective relationships between healthcare providers and patients.

It should be noted that the Turkish Ministry of Health did not provide specific training for contact tracing teams on sociocultural differences or social determinants of health. Pre-pandemic biomedical education also offered limited preparation for fostering cultural competence and humility. Bahar-Özvarış et al. [52], for example, report finding no “specific lectures, sessions, or practices related to intercultural sensitivity and cultural competency” in the medical school curricula they surveyed in Türkiye. Although interest in cultural competence has grown, studies often focus on adapting internationally developed instruments to the Turkish context [52–54], with emphasis on cultural competence toward immigrants or minority groups rather than broader forms of cultural competence. During the COVID-19 pandemic, this absence of training likely constrained health professionals’ capacity to fully interpret and contextualize the sociocultural dimensions of non-compliance.

### Missed opportunities

Against this background, the pandemic response in Türkiye reveals two missed opportunities:

First, the extensive involvement of health professionals in contact tracing constituted a rare experiential encounter with the social determinants of health. However, because of crisis urgency and lack of guidance, there was a limited opportunity to fully integrate a biosocial approach into practice or institutionalize learning from these encounters; consequently, the learning potential of this remained largely informal.

Second, the effectiveness of response measures could have been strengthened by systematically incorporating place-based insights from tracing into prevention strategies. Health professionals identified contextual barriers–from topographical and infrastructural challenges to administrative and socioeconomic vulnerabilities–that shaped the feasibility of timely intervention, yet these observations were rarely integrated into policy-level decision-making. This disconnect can be partly due to the centralized management of the pandemic in Türkiye [55], which prioritized speed and procedural uniformity over the incorporation of place-specific knowledge into planning.

This gap between frontline insight and policy uptake is not unique to Türkiye. Richard et al. [56] similarly argue in their case study of France that, despite the “rediscovery” of social inequalities in health during the pandemic, these factors were not effectively incorporated into pandemic response efforts, and biomedical approaches continued to dominate. Gagnon-Dufresne et al. [2] reached a similar conclusion in their comparative study of contact tracing, noting that, in the four countries they examined, “the biomedical logic dominated the public health responses to COVID-19” particularly in the early stages. Across studies, a key lesson from the pandemic—namely, the importance of social determinants of health—has been emphasized as needing to be incorporated into both medical education and public policy [57, 58]. Local and national health administrations must remain attuned to health disparities and tailor interventions to the specific needs of communities, rather than relying on a one-size-fits-all approach, even during health emergencies [59].

### Contributions of the study

Although most countries employed some form of contact tracing as part of their pandemic response, Türkiye’s nationwide mobilization of diverse health professionals beyond their usual work settings created a unique opportunity to examine place through health professionals’ experiences. Beyond confirming the role of place as a determinant of health and health inequalities, this natural experiment enabled an analysis of the responsiveness of the health workforce and the health system to socioenvironmental drivers. The study identifies key lessons for future public health emergencies. Its findings have implications beyond pandemic management, including professional development and place-sensitive policy making.

### Limitations of the study

While this study focused on health professionals in İstanbul, the findings are likely to be relevant to other urban areas in Türkiye. Contact tracing teams and procedures were centrally designed and implemented nationwide, suggesting that similar dynamics may be observed elsewhere, even though local conditions—especially urban–rural differences and city-specific factors—may affect how these dynamics appear.

## Conclusions

Place fundamentally structures lived experience and can be fully apprehended only through embodied presence. The spatial, social, and environmental characteristics of place shape how citizens experience health and illness in everyday life, while health professionals’ usual experience is largely disconnected from this reality.

The COVID-19 pandemic, particularly through home-based contact tracing, has created a unique experiential window through which health professionals encountered where health originates. Health professionals encountered the complexities of place, including topographical, developmental, and urban planning challenges, alongside the administrative underappreciation of place. They observed firsthand the intricate interplay between socioeconomic and environmental conditions and health. They felt distressed by the socioeconomically deprived households, as these embodied realities challenged their skills and capabilities. At times, they were ambiguous, and at others, judgmental about the sociocultural factors they perceived as straining the effectiveness of biomedicine, reflecting gaps in cultural competence. They recognized the limitations of a purely biomedical approach and the significance of social determinants in health disparities. However, the immediate demands of crisis management often overshadowed the integration of these insights into practice, resulting in missed opportunities for both professional development and the effectiveness of the pandemic response. Decontextualized policies tend to overlook this experience of place, creating a gap between standardized procedures and lived reality. Yet this large-scale natural experiment showed that attending to place offers a way to reorient professional practice and health services.

The findings of this study have implications for professional development, emergency management, and health services beyond. *Health professionals* should be equipped with skills to address socioenvironmental factors shaping people’s health. In particular, the cultural competence of health professionals should be improved through continuous learning. *In future health emergencies*, local health administrations should tailor the interventions according to the changing needs of different places across the city. This requires decentralized management and continuously incorporating insights from the field. *Beyond emergencies*, health authorities should acknowledge place as a source of health and health inequalities. They should provide health professionals with the means to address socioenvironmental factors in their daily practice and create opportunities for them to gain experience with relevant place-based practices free from time-pressure and high stakes.

## Supplementary Information

Supplementary Material 1.

## Data Availability

The datasets generated and/or analyzed during the current study are not publicly available due to the highly sensitive and personal nature of the interviews. Sharing the data would compromise participant confidentiality, as guaranteed in the informed consent process.

## Abbreviations

COVID-19: Coronavirus disease 2019
SARS: Severe acute respiratory syndrome
SES: Socioeconomic status
WHO: World Health Organization
TURKSTAT: Turkish Statistical Institute
DGM: Directorate General for Mapping
