# Supplementary material for: Stepping outside the clinic walls: health professionals’ encounters with place during the COVID-19 pandemic
Source: BMC Public Health. 2026 Mar 26;26:1459. doi: 10.1186/s12889-026-27092-y (PMC13141422; doi:10.1186/s12889-026-27092-y)
Supplement: Supplementary file 1 — Supplementary Material 1. [file 12889_2026_27092_MOESM1_ESM.pdf]

Semi-Structured Interview Form

Demographic Questions:

- Profession (and specialization, if any):
- Age:
- Years of professional experience:
- Duration of professional practice in Istanbul:
- How long have you been working in the contact tracing team? Which unit did you work in previously?
- Had you conducted contact tracing prior to the COVID-19 pandemic?

1. Could you describe the contact tracing activities?
    a. What kinds of activities are conducted within the scope of contact tracing?
    b. Could you describe your own duties and responsibilities within the contact tracing team?
    c. Are there standard procedures applied during contact tracing activities? If yes, what are they? Are there situations in which you have had to deviate from these standard procedures while working in the field? Could you provide examples?
    d. How did you learn how contact tracing is carried out?

2. What does contact tracing mean to you?
    a. Where do you position contact tracing within pandemic management?
    b. Various measures were taken within the scope of COVID-19 pandemic management, such as evening or weekend curfews. Could you compare these measures with contact tracing activities?
    c. Could you compare contact tracing activities with hospital services during this period?

3. Could you describe how contact tracing teams are organized?
    a. Who are the members of a contact tracing team?
    b. How are your working hours determined?
    c. Who organizes the teams?
    d. Is there a division between "field" teams and "coordination" teams? If so, how is the division of labor between these teams determined, and based on what criteria?

4. Could you describe the internal relations within your contact tracing team?
    a. How is task distribution carried out within the team?
    b. Do you experience problems within the team? Could you provide examples?
    c. What factors contribute to your team's ability to conduct its work effectively? Could you provide examples?

5. Could you compare contact tracing teams in different districts?
    a. What are the similarities and differences between contact tracing teams?

6. If there were changes made to the procedures implemented in contact tracing activities during the pandemic, could you describe them?
    a. What types of changes were made, and why?
    b. How do you evaluate these changes?

7. Are the activities of contact tracing teams monitored? If so, how?
    a. By whom are they monitored?
    b. According to which criteria—quantitative and/or qualitative?
    c. Are any sanctions imposed? If so, how?

8. Could you describe your experience with contact tracing?
    a. What did you experience?
    b. Could you talk about the positive and negative experiences you had?
        i. What aspects did you find most challenging?
        ii. What aspects were you most satisfied with?
    c. What kinds of reactions did you encounter from patients or their contacts during your visits?
        i. Expressions of satisfaction?
        ii. Complaints?
        iii. Do patients contact you again afterward? If so, for what reasons?

9. What did being a member of a contact tracing team during the pandemic mean to you?
    a. What did you think?
    b. What did you feel?
    c. How were you affected?
        i. Professionally?
        ii. In terms of family life?
        iii. Personally?

10. Based on what you shared during the interview, I understand that you worked in contact tracing activities for approximately … months. Looking back, what do you remember about your contact tracing activities?
    a. Could you describe any striking experiences that remain in your memory? Did you experience anything you believe you will never forget? If yes, what?
    b. Did you share your contact tracing experiences with others? Did you keep notes? For example, did you keep a diary or share your experiences on social media?

11. During contact tracing, did you make any social observations regarding the population in your district? If so, how?
    a. Did you observe the effects of social, economic, or cultural factors on the transmission of the disease? If yes, how? Could you provide examples?
    b. Did you observe social, economic, or cultural factors that affected patients' treatment and isolation processes? How? Could you provide examples?

12. How do you evaluate contact tracing activities in Turkey?
    a. In which respects do you think contact tracing activities have been effective in combating the pandemic?
    b. In which respects do you think they have been insufficient?

13. If you have any suggestions regarding contact tracing activities, could you share them?
    a. In which respects should contact tracing practices be revised to ensure more effective implementation? What kinds of changes should be made?

b. Do you have any suggestions regarding practical tools developed within the scope of COVID-19 contact tracing, such as applications, modules, or guidelines?

14. If there is anything about your contact tracing experience that I did not ask about but you would like to share, I would be glad to hear it.
